# The impact of perioperative anesthesia management-induced immunosuppression on postoperative cancer recurrence and metastasis: a narrative review

**DOI:** 10.3389/fonc.2025.1558652

**Published:** 2025-08-13

**Authors:** Yaxin Teng, Yanfang Yin, Yanan Shi, Junhui Zhao, Meiyan Sun, Xiaoyong Zhao

**Affiliations:** ^1^ School of Anesthesiology, Shandong Second Medical University, Weifang, Shandong, China; ^2^ Weifang Hospital of Traditional Chinese Medicine, Shandong Second Medical University, Weifang, Shandong, China; ^3^ Department of Anesthesiology, Shandong Cancer Hospital and Institute, Shandong First Medical University and Shandong Academy of Medical Sciences, Jinan, Shandong, China

**Keywords:** perioperative period, anesthesia management, general anesthesia, regional anesthesia, cancer metastasis, cancer recurrence, immunosuppression

## Abstract

Perioperative anesthesia management not only ensures safe and smooth surgery, but its potential immunomodulatory function has also triggered close attention from many researchers. Surgical/anesthetic drugs can cause immunosuppression characterized by decreased natural killer (NK) cell activity, suppression of helper T cell (Th1) function, and imbalance of pro-inflammatory factors. The immunosuppressive microenvironment allows residual cancer cells to evade recognition by the host immune system, resulting in proliferation and distant metastasis. Several retrospective studies have demonstrated an association between cancer patients receiving inhalation anesthesia and reduced recurrence-free survival compared with cancer patients receiving propofol anesthesia. Regional anesthesia techniques may reduce the risk of postoperative recurrence of certain cancers by reducing the amount of systemic opioids and mitigating surgical stress, which in turn may reduce the risk of recurrence after surgery. This review also discusses the effects of pain, blood transfusion, hypothermia, blood pressure, and psychological stress on postoperative metastatic recurrence and immune function in cancer patients. However, observational studies of cancer outcomes after radical surgery for many cancer types under different anesthesia techniques have reported conflicting results, and large, prospective, randomized clinical trials (RCTs) are needed to clearly optimize anesthesia strategies, and to provide new ideas for future efforts to minimize immunosuppression and improve the long-term survival of cancer patients through individualized anesthesia regimens.

## 1 Introduction

In 2022, global cancer statistics released by the International Agency for Research on Cancer (IARC) show that there are nearly 20 million new cancer cases worldwide, along with nearly 10 million cancer deaths. Based on demographics, the number of new cancer cases per year is projected to soar to 35 million by 2050, a 77% increase from 2022. This dramatic increase is a wake-up call for the global public health system (1). Metastasis is responsible for the death of more than 90% of cancer patients, and the occurrence of metastasis is closely related to the body’s immune function (2, 3). Surgery is the main method for removing the primary cancer and metastatic lymph nodes. In fact, some cancer cells may remain after surgery and may proliferate in the parenchyma of organs and tissues through lymphatic and vascular dissemination during the surgical procedure, a process explained in detail by the “seed and soil” hypothesis proposed by Paget in 1889 (4, 5). The perioperative period is centered around the entire surgical procedure and encompasses the preoperative, intraoperative, and postoperative periods. Immunosuppression resulting from the surgery itself, anesthesia, pain, and other perioperative factors has been shown to be a well-established phenomenon (6). The progression of cancer recurrence is influenced by the interplay between two critical factors: the metastatic potential of malignant cells and the anti-metastatic immune activity of the organism (5). The interaction of the two factors determines the outcome of the metastatic process (7).

**Figure 1:**
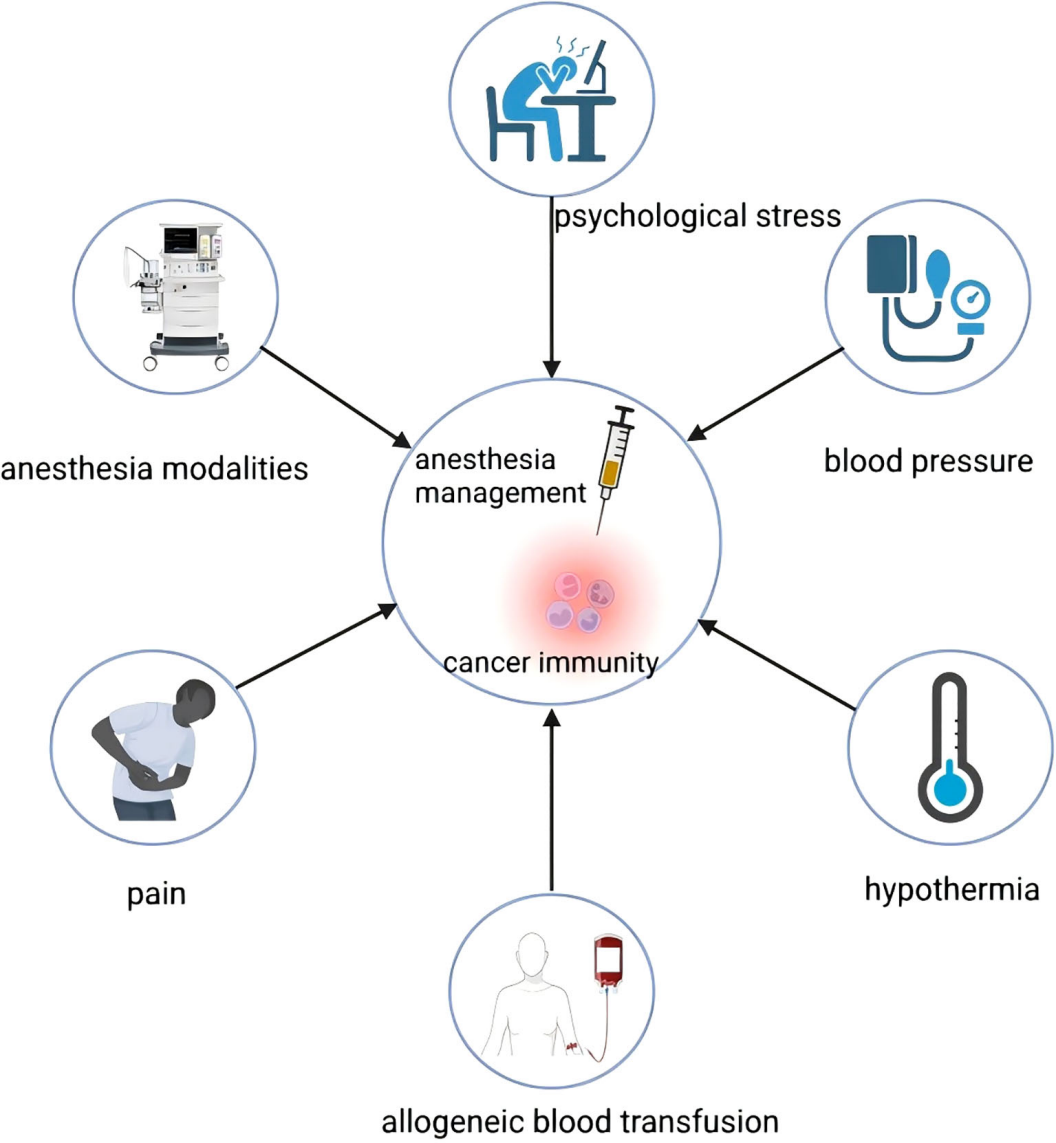
Some factors such as anesthesia modalities, blood transfusion, pain, hypothermia, blood pressure and psychological stress in the perioperative period have an impact on the immunity of cancer patients.

Surgery for cancer patients requires anesthesia, and the type of anesthesia and drugs used can influence the patient’s postoperative stress response, inflammation, immune response, and cognition after surgery. Importantly, both anesthesia and surgery impair the host’s immune function (8). Immunosuppression in cancer patients varies depending on the anesthesia techniques and anesthetic drugs used during anesthesia management (9, 10). The elevated mortality rate observed after cancer surgery highlights the need to explore strategies for minimizing the risk of cancer recurrence. Recent researches suggest that anesthesia management may serve an important purpose in this regard. Individualized anesthesia management protocols may positively impact surgical outcomes in cancer patients, including the management of various intraoperative physiological factors such as pain (11), blood transfusion (12), temperature (13), blood pressure (14) and psychological stress (15) (**Figure 1**). Considering all factors, the perioperative period is essential in shaping the long-term outcomes for cancer surgery patients. This paper examines how perioperative anesthesia management influences immunomodulation, cancer recurrence, and metastasis in cancer patients, with particular emphasis on the progression of metastatic cancer. Additionally, we propose strategies for managing cancer patients during the perioperative period. The authors used the following search strategy in the PubMed database:(anesthesia)AND(cancer metastasis or recurrence)AND(immunization or immunosuppression). Relevant articles and reviews from the last 15 years were manually searched for eligible studies.

## 2 Immunoregulatory mechanisms in cancer patients

The body’s anti-cancer immune response is governed by a complex signaling network, with the body’s cellular immunity considered the main defense against cancer. It is essential in safeguarding against cancer invasion. Adaptive immune effector cells, including CD_8_^+^ cytotoxic T lymphocytes (CTLs), CD_4_^+^ Th1 cells, and innate immune cells such as NK cells, macrophages, and dendritic cells, play key roles in the immune response. Among them, the anti-cancer effects of CTLs and Th1 cells are particularly crucial. CTLs are the main effector cells, primarily killing cancer cells through the perforin-granzyme pathway and the death receptor pathway. CD_4_^+^ Th cells assist in activating CD_8_^+^ CTLs and also produce cytokines that indirectly contribute to the anti-cancer immune response, common pro-inflammatory cytokines include IL-1, IL-6, IL-8, and tumor necrosis factor-α (TNF-α), while common anti-inflammatory cytokines include IL-4 and IL-10. NK cells are the first barrier against cancer, and can kill target cells through four methods (16). Ishigami and co-workers demonstrated that the degree of infiltration of NK cells was associated with the prognosis of patients (17, 18). Growing evidence suggests that NK cells will be pivotal in the treatment of cancer (19). In cancer immunity, macrophages can have both beneficial and harmful effects. On the positive side, macrophages can act as antigen-presenting cells to present tumor antigens and trigger specific immune responses, or indirectly kill cancer cells. On the negative side, macrophages can be reprogrammed by factors secreted by cancer cells to become immunosuppressive tumor-associated macrophages, which can promote cancer development. The stress from anesthesia and surgery during the perioperative period actives the HPA axis and the SNS. This neuroendocrine response increases the levels of soluble immunosuppressive factors and reduces the activity of NK cells and CTLs (20).

## 3 Impact of anesthesia management on immune function

### 3.1 Effects of anesthesia modalities on immune function

#### 3.1.1 General anesthesia

General anesthesia involves delivering anesthetics via inhalation, intravenous, or intramuscular methods, resulting in a temporary suppression of the central nervous system. The clinical effects include loss of consciousness, absence of generalized pain sensation, anterograde amnesia, reflex inhibition, and skeletal muscle relaxation.

Currently, the most commonly used clinical approaches to general anesthesia include inhalational anesthesia, intravenous anesthesia, and combined intravenous-inhalation anesthesia. Studies have found that these methods and drugs significantly affect patient stress responses, inflammation, anti-cancer immunity, cancer progression, and survival in the long term. The most commonly used intravenous and inhalation anesthetics are propofol and sevoflurane, respectively. Clinical evidence shows that propofol may reduce cancer recurrence compared to inhalational anaesthetics (21). Studies have shown that propofol enhances CTL activity, reduces the production of pro-inflammatory factors, and does not affect Th1/Th2, CD_4_^+^/CD_8_^+^, or IL-1/IL-4 ratios. The level of hypoxia inducible factor-1α (HIF-1α) in cancer is inversely proportional to the prognosis of patients (22), and propofol can inhibit the translation process of mRNA, thus inhibiting the activity of HIF-1α in cancer cells in a hypoxic environment, which in turn inhibits angiogenesis and cancer cell proliferation and metastasis. Benzonana and co-workers exposed renal cell carcinoma cells (RCC4) to 0.5-2% isoflurane and found that HIF-1α production was dose-dependently induced and HIF-1α expression was induced through the PI3K/Akt pathway (23). Inhalation anesthetics impact both the central nervous system and the immune system (24). Isoflurane, sevoflurane, and halothane, which are inhalation anesthetics, decrease cytotoxicity in NK cells, while sevoflurane triggers apoptosis and increases HIF-1α expression in T lymphocytes (25, 26). Inhalation anesthetics lead to increased expression of proteins like vascular endothelial growth factor A, matrix metalloproteinase 11(MMP11), transforming growth factor-β (TGF-β), and C-X-C motif chemokine receptor 2, which are linked to cancer growth, migration, and metastasis, in ovarian cancer cells (27).

Presently, there is considerable controversy surrounding propofol’s impact on cancer prognosis. Anti-inflammatory properties of propofol may be linked to improved postoperative survival in cancer patients (28). In a prospective research conducted by Markovic-Bozic et al., patients undergoing craniotomies had significantly higher concentrations of IL-10 when propofol was used as compared with sevoflurane (29), and IL-10 inhibited cancer metastasis by increasing NK cell activity. In animal models, certain anesthetics, including ketamine, sodium thiopental, and volatile agents, reduce NK cell activity, thereby increasing the risk of cancer metastasis. In contrast, propofol does not impair NK cell cytotoxicity (29). Total intravenous anesthesia (TIVA) with propofol in breast cancer surgery reduces the risk of recurrence five years after modified radical mastectomy compared with sevoflurane, a retrospective study shows (30). Jun et al. conducted a retrospective study on esophageal cancer patients and discovered that using propofol anesthesia during surgery improved both overall survival and recurrence-free survival compared to volatile anesthetics (31). Conversely, there were no notable differences in postoperative survival or recurrence rates between propofol-based TIVA and sevoflurane for patients with non-small cell lung cancer (32). Similarly, Enlund et al. conducted a five-year follow-up on a pragmatic randomized controlled trial involving breast cancer patients and discovered no significant difference in overall survival between those who received propofol and those who received sevoflurane general anesthesia (33). Hasselager observed a weak relationship between inhalation anesthesia and colorectal cancer recurrence compared to intravenous general anesthesia, and no relationship between the two anesthesia modalities in terms of overall mortality or disease-free survival rates (34). In addition, a clinical study that recruited older adults requiring major cancer surgery compared the incidence of delayed postoperative neurocognitive recovery under sevoflurane-based and propofol-based general anesthesia, and showed a low incidence in the propofol group (35). Most data comparing volatile anesthetics with TIVA come from *in vitro* or retrospective studies. There are no definitive conclusions about how propofol affects prognosis in different types of cancer, but the data show a trend toward favoring propofol.

Dexmedetomidine is a highly selective α2-adrenergic agonist with sedative, analgesic, anxiolytic and anti-stress effects. Studies have shown that dexmedetomidine can effectively reduce the inflammatory response triggered by postoperative stress, alleviate postoperative immunosuppression in cancer patients, and improve overall immune function. It decreased postoperative levels of C-reactive protein (CRP), TNF-α, and IL-6, while increasing levels of IL-10. In addition, it increases the number of NK cells, B cells, CD_4_^+^ T cells, and the CD_4_^+^/CD_8_^+^ ratio (36). However, dexmedetomidine exhibits different effects in cellular biological behavior depending on the cancer cell type (37). Dexmedetomidine application in cancer patients is still common, and it has an excellent role in reducing postoperative delirium in particular. Due to the lack of strong clinical evidence, it is not possible to draw firm conclusions about whether dexmedetomidine may have an effect on cancer recurrence.

Ketamine is an N-methyl-D-aspartate (NMDA) receptor antagonist with agonistic properties on μ and δ opioid receptors. Ketamine modulates immune function through three main mechanisms: First, ketamine inhibits the expression of pro-inflammatory cytokines (e.g., IL-6 and TNF-α) in the early postoperative period, which mediates anti-inflammatory effects. Second, similar to other analgesics, ketamine significantly inhibits the cytotoxicity of NK cells, which in turn increases the susceptibility to tumor metastasis. Third, ketamine disrupts the homeostasis between different T-cell subpopulations, suppressing antitumor immune function in a dose-dependent manner, and consequently is associated with an increased risk of cancer recurrence and reduced patient survival (38). However, Cho et al. found that intraoperative low-dose ketamine administration did not have any beneficial effects on NK cell activity, inflammatory markers (IL-6, CRP, TNF-α), or 2-year cancer recurrence did not produce any favorable effects (39). In summary, there is a lack of sufficient scientific evidence for the perioperative use of ketamine to improve the prognosis of cancer patients.

#### 3.1.2 Regional anesthesia

Regional anesthesia, including either spinal or paravertebral blocks, can be used alongside general anesthesia or for providing postoperative pain relief (40). Regional anesthesia can help protect the patient’s postoperative immune function and reduce the risk of cancer metastasis and recurrence by blocking harmful nerve impulses from reaching the central nervous system, reducing neuroendocrine responses triggered by surgical stimuli, and lowering the use of volatile anesthetics and opioids during surgery (41). Also, direct absorption of local anesthetics (LAs) inhibits cancer progression (42).

LAs act in association with many signaling pathways and are known for blocking voltage-gated sodium channels, as well as interacting with calcium-potassium and hyperpolarization-gated ion channel ligands gated channels and G protein-coupled receptors. LAs influence both the activation of several downstream pathways in neurons and the structure and function of different membrane types. To access their site of action in neuronal membranes, LAs must cross various tissue barriers (43). Lidocaine is the most commonly used LA, and the most familiar pharmacologic effects of lidocaine are analgesia and antiarrhythmia. In addition, lidocaine possesses interesting anticancer properties, which may be beneficial for long-term cancer treatment outcomes (40). We focus on the anticancer properties of lidocaine. At present, several mechanisms have been suggested to explain the impact of lidocaine on cancer recurrence, which can be generally categorized into five main areas: pathway inhibition, induction of apoptosis, DNA-mediated effects, cell cycle-mediated effects, and reduction of cancer metastasis (44). The epigenetic profile of cancer cells is one of the determinants of the metastatic potential of circulating cancer cells. Epigenetic mechanisms are responsible for regulating methylation of specific regions of DNA, and high methylation levels can be oncogene inactivation leading to cancer progression. Studies suggest that lidocaine, at concentrations relevant to clinical use, demethylates breast cancer cells *in vitro* in a dose-dependent fashion. Furthermore, its effects are potentiated when combined with the chemotherapy agent 5-aza-2’-deoxycytidine (DAC) (45, 46). Wei and others conducted animal experiments and found that lidocaine not only effectively inhibited the progression of hepatocellular carcinoma cells, arresting cells and inducing apoptosis in the G0/G1 phase of the cell cycle and also improved the sensitivity of hepatocellular carcinoma cells to cisplatin *in vivo*, providing a new treatment strategy for the therapy of hepatocellular carcinoma (47). Epidermal growth factor receptor is a transmembrane glycoprotein located on the surface of cell membranes, when it binds to a ligand, can activate the mitogen-activated protein kinase (MAPK) pathway, regulated by the extracellular signal-regulated kinases (ERK1/2), and p38 pathway. Lidocaine and bupivacaine can induce apoptosis in thyroid cancer cells through the MARK signaling pathway (48). Wei et al. conducted treatment of hepatocellular carcinoma cells with lidocaine, and the immunoblotting analysis revealed a reduction in B-cell lymphoma-2 (Bcl-2) levels, alongside an increase in Bcl-2-associated X protein (Bax) levels (47). The balanced relationship between pro-apoptotic mediators (such as Bax) and anti-apoptotic mediators (such as Bcl-2) in human cells determines whether programmed cell death occurs in damaged or pre-cancerous cells.

Retrospective studies have frequently highlighted the potential importance of regional anesthesia in lowering the risk of cancer recurrence following surgery for breast, colon, and prostate cancer patients. In a trial with 1,583 female patients with early-stage breast cancer showed that administering a peritumoral injection of 0.5% lidocaine 7 to 10 minutes prior to surgery resulted in improved disease-free survival and overall survival (49). The combination of general anesthesia and epidural anesthesia has been found to improve overall survival in colorectal cancer patients, particularly those with colon cancer, when compared to general anesthesia alone, according to a recent meta-analysis (50). A large retrospective analysis suggested that regional anesthetic techniques may be beneficial for cancer outcomes after prostate cancer surgery (51). In addition, regional block anesthesia may affect the expression of several cytokines expressed perioperatively, decreasing the release of IL-6 and relatively maintaining the levels of IL-2 and IL-10 (52), and may maintain NK cell activity. Nevertheless, Daniel et al. discovered that regional anesthesia-analgesia was not more effective than volatile anesthetics and opioids in reducing breast cancer recurrence after potentially curative surgery (53). Paul et al. conducted a randomized controlled trial of patients undergoing abdominal surgery for cancer and showed that the use of intraoperative epidural blocks was not associated with improved cancer-free survival (54). Since most of the current clinical findings are based on retrospective studies, there is still an ongoing discussion about how regional anesthesia impacts the outcomes of cancer patients in clinical settings.

### 3.2 Pain

Pain is an uncomfortable experience, both sensory and emotional, that is connected to actual or potential damage to tissues. Globally, cancer is the primary cause of mortality, with pain being a frequent accompanying symptom. It significantly impacts patients’ physical and psychological well-being and also increases the mortality associated with many types of cancer (55). In addition, the immune system and pain are closely related (56). Pain in cancer patients can be acute or chronic. Perioperative acute pain is the result of surgical trauma, inflammation and hyperactivity of the sympathetic nervous system, the latter being a major cause of the transformation of acute pain into chronic and persistent postoperative pain (57). Acute pain lasts for 6 months or less and then subsides. Chronic pain may be related to the cancer itself, cancer treatment, or caused by another condition unrelated to cancer (58).

NK cells are thought to be suppressed by acute pain and their cytotoxic activity is reduced in animal models, increasing the risk of cancer metastasis and recurrence. Pain can generate a stress response that activates the hypothalamic–pituitary–adrenal axis (HPA) and the sympathetic nervous system (SNS), which in turn causes a cascade of immunosuppression. This neuroendocrine response can lead to an increase in beta-endorphin levels in immune cells within the peripheral immune system (59–61). In a clinical trial conducted by Yoon and others (62), they meticulously investigated the alterations in NK cell activity and cellular subsets within the peripheral blood of individuals suffering from chronic pain. Their findings revealed that the cytotoxic activity of NK cells among chronic pain patients did not deviate significantly from that observed in normal patients. Massart et al. concluded (63) that chronic pain alters DNA in the brain and immune system. The origin of cancer pain is partly attributed to tissue damage and inflammation in the tumor microenvironment, though the precise mechanisms are not yet known (64). A recent study has pinpointed macrophage-to-neuron-like cell transformation as a direct mechanism contributing to cancer pain, potentially serving as a therapeutic target for addressing this condition (55). Evidence suggests that postoperative pain management has an impact on surgical outcomes and can reduce cardiac, pulmonary, and metabolic complications (65). Beilin et al. (66) assessed the impact of three postoperative pain relief techniques on immune function, finding that patients with patient-controlled epidural analgesia experienced considerably less pain after surgery, reduced inhibition of lymphocyte proliferation to mitogen, and reduced pro-inflammatory cytokine response to surgery. Further investigation by the team revealed that using preemptive epidural analgesia reduced postoperative pain and lowered the levels of pro-inflammatory cytokines (67). Other studies have shown that local analgesia reduces surgery-associated immunosuppression, which can lessen the occurrence rate of postoperative infections (68) and the risk of metastasis (69). Perioperative pain management is critical, achieving adequate analgesia during perioperative surgery with the least amount of side effects is essential for anesthesiologists.

The use of opioids is the mainstay of anesthesia and perioperative analgesia for patients undergoing cancer surgery. One of the most prominent effects of opioids on the immune system is their ability to inhibit the proliferative process and differentiation of T lymphocytes, while also accelerating the apoptotic process of T lymphocytes. For example, morphine, fentanyl, alfentanil, and sufentanil all decrease NK cell activity, whereas remifentanil has been shown to completely inhibit lymphocyte proliferation and NK cell activity in rats (70). Studies have demonstrated that postoperative analgesia with sufentanil reduces the number of Th17 and regulatory T cells (Tregs) in a surgical model of hepatocellular carcinoma in rats (71). In addition, compared with morphine, sufentanil has less effect on CD_4_^+^/CD_8_^+^ ratio and Treg frequency, making it more suitable for postoperative analgesia. Research evidence on the role of opioids in cancer growth and metastasis is conflicting. It promotes cancer cell invasion and migration through upregulation of MMP in breast and lung cancer, and through upregulation of urokinase fibrinogen activator in colon cancer (24). However, 2 prospective RCTs have found the opposite. In a small trial (n = 146), no differences in biochemical recurrence were observed between opioid-free anesthesia and opioid-based anesthesia in a prostatectomy cohort (72). A prospective, noninferiority RCT comparing sufentanil-based anesthesia versus epidural anesthesia (n = 81 in each group) found that tumor-associated immune alterations between the two groups and cancer-related outcomes (metastasis, recurrence, and survival) did not differ between the two groups (73).

Nonsteroidal anti-inflammatory drugs (NSAIDs) are another commonly used analgesic adjunct in the perioperative period. Cyclooxygenase (COX) is known to convert arachidonic acid into prostaglandins, and the overproduction of prostaglandins has been shown to be critical for various cancer events (74). NSAIDs, as COX inhibitors, reduce prostaglandin synthesis, thereby exerting anti-inflammatory and immunomodulatory effects, as well as affecting immune cell activity and modulating the production and Release (25). 2021 A systematic evaluation published in 2021 (19 studies involving 12,994 participants) found that perioperative use of NSAIDs was associated with longer disease-free survival (HR=0.84 (95% CI, 0.73-0.97)) and overall survival (HR=0.78 (95% CI, 0.64-0.94)), in particularly in patients with breast and ovarian cancer. The authors warned that because most of the included studies were retrospective and highly heterogeneous, the level of quality of these results was low. Two relatively large trials examining extended courses of NSAIDs after initial surgical treatment failed to demonstrate any benefit from long-term exposure to NSAIDs. First, an RCT including 2526 patients with stage 3 colorectal cancer were randomly assigned to receive either celecoxib or placebo combined with fluorouracil, folinic acid, and oxaliplatin (FOLFOX) adjuvant chemotherapy for 3 years. Results showed no difference in disease-free survival between the two groups (75). Another study, which included 2639 patients with ERBB2-negative breast cancer, showed no benefit of celecoxib 400 mg/d for 2 years on 5-year disease-free survival. The results of this study are summarized below (76).

Overall, there is no high-quality evidence that the adjunctive use of NSAIDs and opioids has an impact on cancer outcomes, and higher-quality clinical evidence in the form of prospective randomized controlled trials is needed.

### 3.3 Allogeneic transfusion

Cancer surgery involves extensive resection, and the availability of intraoperative blood transfusion depends on a number of confounding factors, including the degree of the patient’s preoperative anemia and the complexity and difficulty of the procedure (77). There is no doubt that blood transfusions can be life-saving when clinically indicated. Preoperative anemia, intraoperative bleeding, allogeneic transfusion and postoperative anemia can individually or collectively affect the long-term prognosis of cancer patients. This article focuses on the effects of allogeneic blood transfusion. Studies have shown that transfusion-induced transient immunosuppression may be linked to poor prognosis in cancer patients (78). Transfusion-related immunomodulation refers to the immunosuppressive effects associated with allogeneic blood transfusion. It occurs by interfering with the activity of CTLs and monocytes, decreasing the production of immune cytokines (e.g., IL-2, IFN-γ), and increasing the activity of suppressor T cells, which promotes the release of prostaglandins. Besides residual leukocytes, concentrated red blood cells contain bioactive substances, which generally have immunosuppressive and cancer-promoting effects (79).

In addition, the storage time of blood products had been of great concern. L-arginine is required for T-cell activation and proliferation, and high levels of free arginine after transfusion may underlie immunosuppression and transfusion-associated infections. Despite its short half-life, arginine may be involved in early immunosuppression. Thus, elevated levels of free arginine in long-stored blood may have implications for immunocompromised patients. Experimental data from Mollinedo showed that free arginine levels in concentrated red blood cell units increased with storage time (80). Using a rat model of erythrocyte storage and transfusion, Hod et al. showed that transfusion of stored erythrocytes or washing of stored erythrocytes increased plasma non-transferrin bound iron, leading to acute iron deposition in tissues and triggers inflammation (81). This is a concern in immunosuppressed cancer patients, and the storage time of the blood product being transfused should be considered when transfusing blood to cancer patients. The benefits of transfusing concentrated red blood cells with a short storage time outweigh the drawbacks. The study by Kekre et al. involved 27,000 cancer patients treated with radiochemotherapy or surgery, of whom 1,929 received transfusion therapy. The results showed that the storage time of transfused red blood cells had no effect on OS or cancer recurrence (82). In conclusion, these results highlight the significance of limiting blood product use in malignant cancer patients, supporting a stricter transfusion threshold (83).

Supported by several meta-analyses, blood transfusions do have adverse effects on outcomes for many types of cancer. Pang et al. pooled 34 observational clinical studies, covering a total of 174,036 patients, which clearly showed that perioperative blood transfusion has a significant opposite forces on long-term outcomes and also augments the risk of short-term complications after colorectal cancer operation (84). Sun et al. in order to find out the relationship between allogeneic blood transfusion and cancer prognosis in patients with gastric cancer who underwent radical surgery, included 18 studies (9,120 patients with gastric cancer), of which 36.3% received transfusions, and they found that receiving allogeneic blood transfusions was linked to increased rates of mortality from all causes, cancer-related deaths, and cancer recurrence (85). Tai and others investigated how blood transfusion affects the prognosis of hepatocellular carcinoma patients. Their analysis revealed that the risk ratio reached its highest point at a transfusion threshold of 5–6 units. Additionally, they observed that autologous blood transfusion had minimal influence on the perioperative humoral immune function in these patients. These findings indicate that autologous transfusion not only minimizes the likelihood of adverse transfusion reactions but also significantly lowers the risk of disease transmission associated with stored blood (86). In situations of massive blood loss, autologous blood transfusion is a proven method. However, the biggest controversy regarding autologous transfusion in the context of cancer is that metastasis caused by cancer resection may lead to systemic dissemination following autologous transfusion. However, to date, the evidence for this theory is limited. Consequently, the use of autologous transfusion as an alternative to allogeneic transfusion in managing cancer patients during surgery requires more exploration (78).

### 3.4 Hypothermia

Body temperature is considered a vital sign, alongside blood pressure, heart rate, and breathing rate (87). In general, intraoperative hypothermia occurs in more than 50% of surgical procedures (13), and impaired control of normal thermoregulation induced by anesthetics is the primary cause of hypothermia in most patients (88). Clinical research has revealed that even a small drop in body temperature can lead to major problems, like surgical wound infections (89), coagulation disorders, increased allogeneic transfusions (90) and delayed recovery from anesthesia (91). Intraoperative hypothermia can influence the immune system, potentially affecting cancer recurrence and metastasis post-surgery (13).

T cell subsets are essential indicators for assessing immune function in human cells and are vital in the body’s anticancer immune response. The immune system’s effectiveness in battling cancer is greatly reduced by an imbalance in the amount or function of these cells and the cytokines they produce. This disruption can, in turn, reduce the efficacy of cancer therapies and negatively impact patient prognosis (92). The type 1 adaptive immune response, which is mediated by Th1 and CTL cells, is considered an important component of immunity against cancer cells (93). In contrast, Treg cells and Th2 cells are considered to be the two main T cells that nullify the anticancer immune response (94). Du et al. showed (95) that hypothermia significantly contributes to the immunosuppressive microenvironment in cancer patients. This condition results in an expansion of splenic Treg and Th2 cell populations, elevated levels of IL-4 and IL-10, and an increase in local hypothermic Treg cells along with higher TGF-β1 concentrations in the cancer, thereby facilitating lung metastasis. In this model, ischemic conditions within the cancer lead to local hypothermia, which can trigger a shift in the polarity of the immune response from type 1 to type 2. This shift in turn creates an immunosuppressive microenvironment that effectively protects the cancer from rejection by the immune system. In addition, hypothermia activates the SNS, which in turn prompts the adrenal glands to release catecholamines and small amounts of glucocorticoids. These neuroendocrine responses further inhibit NK cell activity (13, 96), directly or indirectly weakening cell-mediated immunity (97). Neutrophils have an immunosurveillance role against cancer cells. The oxidative function of neutrophils is a crucial factor in defending against infections in surgical wounds. However, the production of reactive oxidative intermediates is linearly correlated with body temperature, intraoperative hypothermia not only reduces the phagocytic capacity of neutrophils, but also reduces the production of reactive oxygen species intermediates, which reduces the body’s resistance to infection (98). Experiments by Seki and co-workers exposed an immunologically active C57BL/6 mouse model of implanted rectal carcinoma to 4°C, and observed an inhibition of cancer growth of up to 80%. Remarkably, similar results have been observed in many mouse models of different types of cancer, indicating the potential of this approach in treating a wide range of malignancies. Following cold exposure, circulating blood glucose levels decrease, and glucose uptake by tumors is reduced, thereby limiting the primary energy source for cancer cells. Zhang and others’ study on breast cancer found that mild hypothermia attenuated the chemotaxis of breast cancer cells but had no significant effect on unidirectional migration ability. This suggests that mild hypothermia can be used as an adjunct therapy in combination with surgery to reduce cancer cell adhesion and migration (99). A new study has found that a temperature window can be identified in a rat model where cell division can be safely halted, and has termed this range “cytostatic hypothermia”. “Cytostatic hypothermia” prevents the growth of glioblastoma in rats, and this study proposes a non-cryogenic hypothermia method that provides a previously unexplored approach to the treatment of glioblastoma (100).

Hypothermia is common in unwarmed patients during surgery. Particularly in patients receiving general or neuraxial anesthesia for longer than 30 minutes, accurate measurement or reliable estimation of core temperature is essential. Unless there are special conditions, it’s important to keep the patient’s core body temperature above 36°C during surgery to ensure both safety and comfort (101).

### 3.5 Blood pressure

The interaction of cardiac output and vascular resistance in the circulatory system results in the body’s arterial pressure, and is characterized by systolic and diastolic components (102). The definition of intraoperative hypotension is still unclear and varies widely (103). The most recent consensus statements and guidelines for managing arterial pressure during surgery suggest keeping the intraoperative mean arterial pressure at a minimum of 60 mm Hg for high-risk patients (102).

Indeed, hypotensive and hypertensive episodes are common during anesthesia and surgery, even when well-controlled, and although the threshold of harm is unknown, a certain degree of hypotension can lead to organ damage, complications, and death (104). In addition, several clinical studies have found that perioperative hypertension or hypotension in cancer surgery may also impact oncological outcomes. In 1991, Younes et.al (105). first reported that a higher number of intraoperative hypotensive episodes correlated with a reduced recurrence-free survival timein patients suffering from liver metastases due to colorectal cancer. On the other hand, perioperative hypertension in individuals with renal or rectal cancer has been identified as an independent risk factor for cancer-specific survival and recurrence-free survival after surgery (106, 107). Huang et al. (108) proposed a new definition for intraoperative hypertension and hypotension, considering their effects on long-term survival. They classified episodes of systolic blood pressure (SBP) exceeding 140 mmHg for a minimum of 5 minutes as intraoperative hypertension, while episodes with SBP below 100 mmHg for at least 5 minutes were categorized as hypotension. After taking into account potential confounding variables, it was found that patients who only had intraoperative hypotension had a much shorter overall survival than those who only had intraoperative hypertension. This study is unclear about the underlying mechanisms through which intraoperative hypotension affects long-term survival, but it suggests that it may be related to (1) intraoperative hypotension increasing the risk of perioperative organ damage, although patient deaths in this study were primarily caused by cancer (2); microenvironmental hypoxia, which is characteristic of solid tumors, and how intraoperative hypotension may worsen hypoxia, promoting cancer invasion and metastasis; and (3) hypoxia caused by intraoperative hypotension potentially increasing systemic inflammation, thereby raising the risk of cancer recurrence and death. Therefore, for anesthesiologists, maintaining stable intraoperative blood pressure in patients with an unstable circulatory system is one of the crucial measures that significantly impact patient outcomes.

### 3.6 Psychological stress

Historically, Western culture has long embraced the concept of mind-body dualism, where the body and mind are viewed as separate entities, sometimes even exhibiting contradictory characteristics. However, despite the emergence of “mind-body medicine” in the twentieth century, it is only recently that it has become more widely recognized that mental and physical health may be deeply interconnected. In particular, the pathophysiological basis of mental illness has reached beyond the boundaries of the central nervous system (15). Understanding how stress and cancer are related is critical, especially in view of the high prevalence of anxiety and depression in those with cancer. According to a meta-analysis (109), 15% of people with cancer have major depression, 20% have minor depression, and 10% have anxiety disorders. Current evidence proves that there is a bidirectional regulatory network between the immune system and the neuroendocrine system (110, 111). The two systems are connected by chemical signals secreted by specific cells, and psychological stress can disrupt these networks (112). The sympathetic-adrenal-medullary (SAM) system and the HPA axis are the two main components responsible for maintaining and restoring homeostasis in the body during stress (113). When stressed, the SAM and the HPA axis are quickly activated, producing catecholamines and glucocorticoids. Neuroendocrine factors linked to stress can directly influence cancer cells’ biological characteristics, including their growth, programmed cell death, and metastatic potential (15). Psychological factors are often overlooked for cancer patients. Stress is closely related to cytokines secreted by cells of the macrophage and monocyte lineages. Under stress, the expression of IL-1, IL-6, and TNF increases significantly, while the expression of IL-2, interferon, and MHC class II molecules decreases. TNF inhibits tyrosine phosphatase activity, and this inhibition further reduces the expression of MHC class I antigens on the surface of cells. Thus, malignant cells are able to evade immune surveillance and create favorable conditions for growth and proliferation (114). While there is no direct proof connecting stress to cancer, extensive epidemiological studies suggest a significant relationship between objectively identified stressors and self-reported psychological distress with negative cancer outcomes. These outcomes include cancer progression, metastasis, recurrence, treatment failure, and an elevated risk of mortality (115, 116).

In clinical studies, effective psychological interventions (PI) are clinically important in helping to improve the psychological quality of patients and thus their quality of life (117). However, the impact of PI on cancer patients is controversial. A systematic evaluation and meta-analysis of randomized clinical trials examining the effects of PI on survival and quality of life (QoL) in cancer patients concluded that PI do not prolong survival, but they can improve patients’ QoL. Their analysis showed that the intervention group showed significant improvements in all four measured QoL domains (holistic, affective, social, and physical) when compared to the control group, with clinical effects in the domain of emotions highest. In terms of cancer type, they found that breast cancer patients benefited the most from PI, and the prostate cancer group did not see improvement in any domain (118). A study by Zhang et al. found that PI benefited QoL and psychological outcomes in colorectal cancer patients (119). Accelerated Rehabilitation Surgery (ERAS) interventions are based on evidence-based medicine, and through a series of measures such as preoperative, intraoperative, and postoperative comprehensive rehabilitation interventions, they can reduce the surgical stress response and promote the rapid recovery of patients. Integrating psychological assessment and intervention into the ERAS process not only accelerates the recovery process, but also improves patients’ psychological status and quality of life, and reduces the incidence of complications.

## 4 Discussion

A growing body of evidence has examined the impact of perioperative anesthetic management on cancer metastasis recurrence and survival in cancer patients. Laboratory studies suggest that the effects of propofol on tumor cell biology, inflammation, and immune function may be more beneficial in preventing recurrence compared to volatile agents. A retrospective study showed an association between propofol TIVA and improved disease-free survival compared with inhalation anesthesia. However, several small RCTs have found no statistical difference between propofol TIVA and inhalation anesthesia cohorts in terms of postoperative circulating tumor cell counts (40). Although many essentially limited retrospective studies have suggested a benefit of propofol TIVA on overall survival, no large RCT has yet to provide data to support this. And data from large RCTs are indispensable before any adjustments to clinical practice can be recommended. A recent meta-analysis included 6 RCTs examining the effect of adjunctive use of RA on cancer recurrence rates in adults undergoing cancer resection. It was concluded that the adjunctive use of regional anesthesia to general anesthesia did not reduce the rate of cancer recurrence during cancer resection surgery (120). However, this finding needs to be interpreted with caution due to the low level of evidence from the included studies, the high degree of heterogeneity, and the potential risk of bias, and more definitive results from the large RCTs are needed. Current research suggests that opioid receptors may be involved in promoting cancer recurrence and migration, and therefore, the development of meticulous genomic analyses of patients’ resected cancer tissues, as well as the exploration of the mechanisms of interactions between individual patients’ cancer gene expression profiles and the status of perioperative opioid use during surgery, as well as subsequent oncological outcomes, has become a new focus of contemporary interest. NSAIDs may exert antitumor effects by exerting an antagonistic effect on inflammation, angiogenesis, and multiple other cellular pathways to produce antitumor effects. However, most retrospective studies and a small number of RCTs have yielded conflicting conclusions. Overall, long-term adjuvant use of these agents has not been shown to have an impact on cancer outcomes, and data from the small number of prospective trials on perioperative NSAIDs are not convincing. There are no definitive conclusions about the effects of body temperature and blood pressure on the prognosis of cancer patients, and there is still a need for large prospective RCTs to provide more definitive information. Immunomodulation associated with blood transfusion in cancer surgery is well documented, but the extent to which it affects cancer progression is unclear. The association between blood transfusion and cancer progression is disease-specific. There is growing evidence that autologous blood transfusions may be safe in cancer surgery. Anxiety and depression are very common in cancer patients, and psychological factors are often overlooked in cancer patients. Therefore, anesthesiologists should try to alleviate patients’ anxiety about surgery as much as possible during preoperative visits and conversations, using medications when necessary. In cytokine-related studies, their concentrations usually exhibit significant interindividual heterogeneity, and this heterogeneity can adversely affect the validity of statistical significance tests. In addition, the variable sensitivity of cytokine assays makes the variability of the generated data increased and noise interference more pronounced.

Although this review summarizes the existing evidence on the potential association between perioperative anesthetic management-related immunosuppression and postoperative cancer recurrence and metastasis, it is important to recognize the significant limitations of research in this field. Firstly, most of the evidence comes from animal models and cell studies. While these allow for detailed examination of the effects of specific anesthetics or anesthetic techniques on immune function under strictly controlled conditions, animal models or *in vitro* cell culture studies inherently differ from humans in terms of immune system complexity, tumor microenvironment, drug metabolism, and disease natural history. Secondly, clinical studies are subject to diverse and complex confounding factors. Factors such as the patient’s underlying medical conditions, the specific type and stage of the tumor, the extent of surgical trauma, the severity of perioperative stress responses, the effectiveness of postoperative pain management, and the use and timing of adjuvant therapies (such as chemotherapy or radiation therapy) can all significantly influence the body’s immune status and tumor prognosis. Since it is difficult to precisely distinguish these confounding factors from the effects of specific anesthetic drugs or techniques, there is no clear evidence that changing a single anesthetic technique can directly impact a patient’s long-term prognosis. Finally, most studies are retrospective and small-scale RCTs, which are inherently limited and cannot serve as the basis for practice changes. Future research requires more rigorous, large-scale prospective RCT studies.

## 5 Conclusion

The intersection of anesthesiology and immunology has stimulated increasing interest, particularly in the exciting possibility that perioperative anesthesia and interventions in oncology patients may meaningfully influence patient prognosis. Current preclinical studies indicate that immunosuppression caused by anesthesia could potentially exacerbate cancer recurrence in patients with specific cancer types. However, the complex relationship between anesthetics, immune response, cancer patient survival, and the occurrence of metastatic recurrence remains unresolved. To provide more conclusive evidence, further prospective randomized controlled trials are essential. Therefore, anesthesiologists should also seek individualized anesthetic regimens that are optimal for their patients.
